# Intraprostatic Fiducials Compared with Bony Anatomy and Skin Marks for Image-Guided Radiation Therapy of Prostate Cancer

**DOI:** 10.7759/cureus.1769

**Published:** 2017-10-12

**Authors:** Juliette Moreau, Julian Biau, Jean-Louis Achard, Ivan Toledano, Charles Benhaim, Fabrice Kwiatkowski, Geneviève Loos, Michel Lapeyre

**Affiliations:** 1 Radiotherapy, Centre Jean Perrin

**Keywords:** fiducial markers, shifts, bony anatomy, prostate, skin marks, margins, image-guided radiation therapy

## Abstract

Purpose

Prostate motion occurs during radiotherapy for localized prostate cancer. We evaluated the input of intraprostatic fiducials for image-guided radiation therapy and compared it with bony anatomy and skin marks.

Methods

Eleven patients were implanted with three fiducial markers in the prostate. Daily sets of orthogonal kV-kV images were compared with digitally reconstructed radiography. Data were recorded for skin marks, bony anatomy, and fiducial markers. The variations were analyzed along three principal axes (left-right: LR, superoinferior: SI, and anteroposterior: AP).

Results

A total of 2,417 measures were recorded over 38 fractions of radiotherapy (76 Gy). Fiducial marker movements from bony anatomy were ≤ 5 mm for 84.2% (confidence interval: CI 95%±1.5), 91.3% (CI 95%±1.1), and 99.5% (CI 95%±0.4) of the measures along the AP, SI, and LR axes, respectively. Ninety-five percent of the shifts between a fiducial marker and the bony anatomy were < 8 mm in the AP and SI axes, and < 3 mm in the LR axis. Fiducial marker movements from skin marks were ≤ 5 mm for 64.8% (CI 95%±1.9), 79.2% (CI 95%±1.6), and 87.2% (CI 95%±1.3) of the measures along the AP, SI, and LR axes, respectively. Bony anatomy movements from skin marks were ≤ 5 mm for 84% (CI 95%±1.4), 92% (CI 95%±1.1), and 87% (CI 95%±1.3) of the measurements along the AP, SI, and LR axes, respectively.

Conclusion

Using fiducial markers provides better accuracy of repositioning of the prostate than using bony anatomy and skin marks for image-guided radiotherapy of prostate cancer.

## Introduction

The use of intensity-modulated radiotherapy (IMRT) for localized prostate cancer enables better covering of target volumes while sparing more of the organs at risk (e.g. bladder, rectum, etc.) [1-4]. Image guided radiotherapy (IGRT) improves the accuracy of the target volume position during treatment. The patient’s set-up can be verified daily on bony anatomy with orthogonal kV-kV portal images. However, bony anatomy verification doesn’t take into account target volume and normal tissue shaping [5]. Several techniques could improve the position verification during external-beam radiotherapy of patients with prostate cancer. Kilovoltage cone beam computed tomography (kV CBCT) is one method to assess and correct for inter-fraction prostate localization immediately before treatment [6]. Another technique which is commonly used is intraprostatic fiducial markers with kilovoltage (kV) orthogonal imaging. This allows strict position verification on the prostate itself.

The aim of the study was to evaluate the position of the prostate with fiducial markers during external-beam radiotherapy and to compare it with bony anatomy and skin marks for image-guided radiation therapy of prostate cancer.

## Materials and methods

Between June and November 2011, eleven patients received IMRT after the insertion of fiducial markers for localized prostate cancer (76 Gy in 38 fractions) using a volumetric modulated arctherapy technique (RapidArc®- Varian Medical Systems, Inc., Palo Alto, CA, USA). The position of the prostate was verified using fiducial markers and orthogonal kV-kV portal images.

Implantation of the fiducial markers

The radio-opaque markers were inserted, under ultrasound guidance, through biopsy needle channels using a transrectal ultrasound probe [7]. Lidocaine was used as local anesthesia. The prophylactic antibiotics, ciprofloxacin and metronidazole, were given before and after the implant procedure (one day before to three days after). Three fiducial markers (Bebig®, Berlin, Germany) were implanted: one at the prostate base, one in the apex in a paraurethral position, and one in the lobes in a peripheral posterior position. The procedure was not carried out for patients with a medical history of prostatitis, recent urinary infection (within less than one month), or those treated with an antiplatelet drug that could not be stopped.

Simulation, treatment planning, and position verification

Two weeks after the insertion of markers, a simulated computed tomography (CT) (GE®, Little Chalfont, U.K.) was carried out with 2.5 mm slice thickness. Skin markers representing patients’ isocenter reference were tattooed on the skin. Patients were asked to empty their bladder and to drink two glasses of water (40 cc) one hour before the CT scan and to empty their rectum. This procedure was repeated each day of treatment [8]. The target volume was outlined to include the prostate and all, or part, of the seminal vesicles. Margins from gross tumor volume (GTV) to clinical target volume (CTV) depended on prognostic factors [9]. The planning target volume (PTV) was generated using a 1 cm margin, except in the posterior direction where a 5 mm margin was applied [9-10]. The organs at risk that were contoured were: the bladder wall, rectal wall, femoral heads, and bulb of the penis. Delineation was performed using the Isogray software (Dosisoft®, Cachan, France)

Digitally reconstructed radiographs were generated from the planning computed tomography scans and used as reference images. Patients were positioned daily according to the reference skin marks. Orthogonal kV-kV electronic portal images were acquired using the Varian On-Board Imager (OBI) software ® (Varian Medical Systems, Palo Alto, CA), and matching was done using bony anatomy. This control was carried out each day of treatment by the technicians and confirmed by a radiation oncologist [11]. Bony control verification was performed to compare it with fiducial position. No shift was applied using these measurements. After each bony control, the position verification was matched to fiducial markers automatically using the OBI software ®, or manually if a failure occurred. A radiation oncologist validated shifts each day from fiducial markers positions. Shifts were measured in the three dimensions: anterior/posterior (AP), superoinferior (SI), and left/right (LR). The displacement measured using fiducial markers was applied for each fraction of the treatment.

Statistics

The setup displacement data were used to calculate the systematic error (Σ), the standard deviation (SD) of the distribution of the average setup displacements per patient, and the random error (σ). These values were used to compare the setup accuracy using skin marks, fiducial markers, and bony anatomy. The data were summarized as a mean systematic error (Σ) (for one patient), a standard deviation of systematic positioning errors (SD) (for the entire population), and a random positioning error (σ). The Σ is defined as the average of the individual mean positions, the SD (for the entire population) is defined as the standard deviation of the individual mean positions, and the σ is defined as the square-root of the average of the individual variances [12].

The median distance and distribution of deviation of absolute differences were compared. In both comparisons, a difference of 5 mm or less was considered acceptable. We also reviewed results using a difference of 3 mm or less (maximum accepted deviation not resulting in a shift). Margins from the CTV to PTV were applied using Van Herk (m =2 .5 Σ + 0.7σ) and Stroom (m=2 +0.7σ) methods [13-14]. We used the Chi 2 test to compare percentages. Differences with p < 0.05 were considered statistically significant.

## Results

All deviations from the planning isocenter were analyzed using the bony anatomy and fiducial markers and were compared with the skin marks. A total of 2,417 measures were recorded.

Fiducial marker movements from bony anatomy

**Figure 1:**
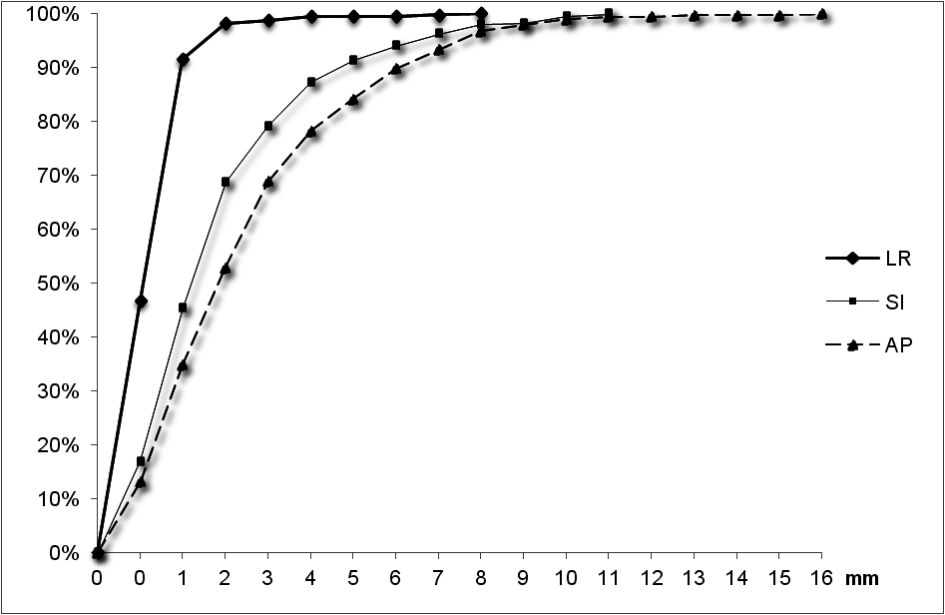
Cumulated percentages of fiducial markers positions compared with bony anatomy using 1208 measures in the three axes. LR: Left-right, SI: superoinferior, and AP: anteroposterior.

The average ± standard deviation (median; maximum) of shifts (in absolute values) were 2.9±2.5 mm (2 mm; 16 mm) in the AP, 2.2±2.1 mm (2 mm; 11 mm) in the SI, and 0.6±0.8 mm (1 mm, 8 mm) in the LR axis. Fiducial marker movements from bony anatomy were ≤ 5 mm for 84.2 % (confidence interval: CI 95%±1.5), 91.3 % (CI 95%±1.1), and 99.5 % (CI 95%±0.4) of measures in the AP, SI, and LR dimensions, respectively (Figure 1).

Ninety-five percent of the shifts between the fiducial marker and bony anatomy were < 8 mm in the AP and SI axes, and < 3 mm in the LR axis. The results are shown in Table 1.

**Table 1:** Fiducial Intraprostatic Movements from Bony Anatomy and Skin Marks on Three Axes for 2,417 Measures For each axe, a Chi 2 test compared results of fiducial markers movements between bony anatomy and skin marks. CI: confidence interval

	Antero-posterior		Supero-inferior		Left-Right	
	Bony anatomy	Skin marks	Bony anatomy	Skin marks	Bony anatomy	Skin marks
≤ 3 mm	68.8% (CI 95± 1.8)	43.2% (CI 95±2.0) p < 10^-6^	79.1% (CI 95±1.6)	60.2% (CI 95±2.0) p < 10^-6^	98.8% (CI 95±0.4)	72.6% (CI 95±1.8) p < 10^-6^
≤ 5 mm	84.2% (CI 95±1.5)	64.8% (CI 95±1.9) p < 10^-6^	91.3% (CI 95±1.1)	79.2% (CI 95±1.6) p < 10^-6^	99.5% (CI 95±0.4)	87.2% (CI 95±1.3) p < 10^-6^

Fiducial marker movements from skin marks

**Figure 2:**
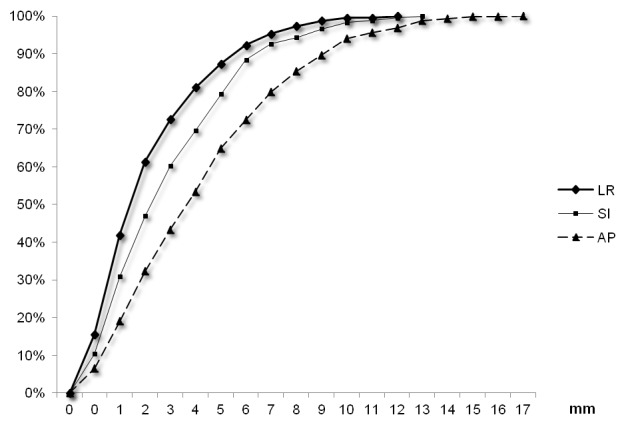
Cumulated percentages of fiducial marker positions compared with skin marks using 1,209 measures in the three axes. LR: Left-right, SI: superoinferior, and AP: anteroposterior.

The average ± standard deviation (median, maximum) of shifts (in absolute values) were 4.9±3.4 mm (4 mm, 17 mm) in the AP, 3.3±2.7 mm (3 mm, 13 mm) in the SI, and 2.6±2.3 mm (2 mm, 12 mm) in the LR axis. Fiducial marker movements from skin marks were ≤ 5 mm for 64.8 % (CI 95%±1.9), 79.2% (CI 95%±1.6), and 87.2% (CI 95%±1.3) of measures in the AP, SI, and LR dimensions, respectively (Figure 2).

Bony anatomy movements from skin marks

**Figure 3:**
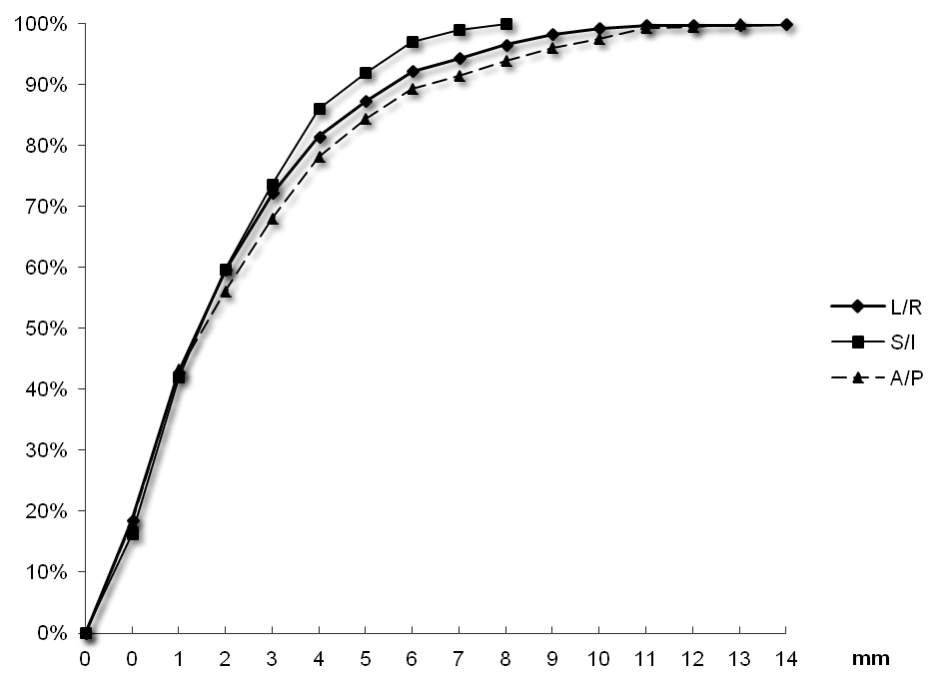
Cumulated percentages of bony anatomy positions compared with skin marks using 1,237 measures in the three axes. LR: Left-right, SI: superoinferior, and AP: anteroposterior.

The average ± standard deviation (median, maximum) of shifts (in absolute values) were 2.8±2.8 mm (2 mm, 13 mm) in the AP, 2.3±1.9 mm (2 mm, 8 mm) in the SI, and 2.7±2.4 mm (2 mm, 14 mm) in the LR axis. Bony anatomy movements from skin marks were ≤ 5 mm for 84% (CI 95%±1.4) in the AP dimension, 92% (CI 95%±1.1) in the SI dimension, and 87% (CI 95%±1.3) in the LR dimension (Figure 3).

Evaluation of systematic errors (Σ) and random errors (σ) to calculate margins

Systematic errors (Σ), random errors (σ), and margins were calculated. Results are displayed in Tables 2 and 3.

**Table 2:** Systematic Errors (Σ) and Random Errors (σ) Calculated for 11 Patients A comparison of fiducial marker deviations (in mm) from bony anatomy and skin marks. AP: anteroposterior; SI: superoinferior; LR: left-right; Σ = the standard deviation of the individual mean positions; σ = the square-root of the average of the individual variances in comparison of systematic errors, test F (comparison of variances)

	Fiducial marker - bony anatomy		Fiducial marker - skin marks	
Errors	Σ (mm)	s (mm)	Σ (mm)	s (mm)
AP	1.4 (p = 0.002)	2.1	4.1	4.1
SI	1.4 (p = 0.04)	1.6	2.8	2.5
LR	0.1 (p < 10^-10^)	0.8	2.7	2.2

**Table 3:** Margins from CTV to PTV on 2,417 Measures According to Van Herk and Stroom CTV: clinical target volume, PTV: planning target volume, AP: anteroposterior; SI: superoinferior; LR: left-right; * 2.5 Σ + 0.7 σ ; **2 Σ + 0.7 σ

	Fiducial marker – bony anatomy		Fiducial marker - skin marks	
	Van Herk* (mm)	Stroom** (mm)	Van Herk (mm)	Stroom (mm)
AP	5	4.3	13	11
SI	5	4	9	8
LR	2	1	8	7

## Discussion

Prostate motion with intraprostatic fiducials seems to allow a more accurate repositioning of patients treated by IMRT-IGRT compared with bony anatomy and skin marks. Ninety-five percent of the shifts between fiducial markers and bony anatomy were < 8 mm in the AP and SI axes and < 3 mm in the LR dimension. However, we have to note that the most significant limitation of the study was the small number of patients (n=11). However, a great number of measures (2,417) were recorded on successive patients by one team.

The shape variation of the prostate was not considered in this study. Deurloo, et al. [15] showed that the measured shape variation was largest at the tip of the vesicles, but remained very small. Therefore, it was a valid approximation in the IGRT of prostate cancer to correct only for setup errors and organ motion. In this series, rotation variations were not studied. Aubry, et al. [16] demonstrated significant rotation variation of the prostate. An analysis of 348 inter-fraction rotations revealed random SDs of 6.1° (systematic SD, 5.6°) around the LR axis, 2.8° (systematic SD, 2.4°) around the SI axis, and 2.0° (systematic SD, 2.2°) around the AP axis. The authors concluded that inter- and intrafraction rotations of the prostate should be taken into account when designing PTV margins because their magnitudes were not negligible. For intrafraction motion of the prostate, Kupelian, et al. [17] demonstrated on 41 patients that displacements > 3 and > 5 mm were observed during 41% and 15% of radiation treatments, respectively.

Budiharto, et al. [18] highlighted the importance of the duration of treatment. In one study, they showed in 27 patients under IMRT treatment that about 21% of the cases, in which treatment lasted longer than 450 seconds, showed prostate displacement > 5 mm at the end of treatment. The use of intraprostatic fiducials as surrogates for the prostate gland position assumed that the markers were rigidly positioned within the prostate. Kupelian, et al. [19], in a large series analyzing 6111 intermarker distances, conclude that the average absolute variation of all inter-marker distances (SD) was 1.01 (1.03) mm. Intraprostatic implanted fiducials in the prostate seem to be a reliable and simple method for localizing the prostate gland, even in the presence of organ deformation. Van der Heide, et al. [20] discussed fiducial migration caused by swelling of the gland during the first treatment week that was then reduced. They concluded that migration due to swelling should not be taken into account. In our center, the CT scan was carried out two weeks after fiducial implantation to avoid this problem. Kumar, et al. [21] concluded that fiducial marker migrations were minimal within the week after implantation (mean distance between fiducial pairs < 1 mm) and that implantation and planning treatment could be performed on the same day. Previous studies reported movement of the intraprostatic fiducials and found systematic errors from 1.1 mm to 4.4 mm in the AP axis and from 1.5 mm to 3.7 mm in the SI axis [20, 22]. Authors also reported results for random errors from 2.4 mm to 3.3 mm in the AP axis and from 2.2 mm to 2.7 mm in the SI axis. Our data fall within this range.

McNair, et al. [23] evaluated margins on 30 patients with three fiducial markers inserted who had pretreatment and post-treatment images acquired and were treated using an offline correction protocol and gold markers. They observed a decrease in the margins when comparing bony anatomy with fiducial markers, from 4.7 mm to 4.3 mm in the LR, from 6.3 mm to 4.2 mm in the SI, and from 8.4 to 6 mm in the AP axis. In our study, similar results are reported (Table 3). However, margin calculations should be based on the inter-fraction motion as well as intrafraction motion and rotations. In the same study, McNair, et al. observed the potential benefit of using fiducial markers with the online correction protocol to determine the presence and effect of intrafraction motion. They concluded that margins could be reduced to 3.6 mm, 5 mm, and 5.6 mm in the LR, SI, and AP directions, respectively. Rijkhorst, et al. [24] reported that with the online CTV translation correction, a 7 mm margin was sufficient.

Others techniques, such as CBCT, can be used to assess and correct for inter-fraction prostate localization immediately before treatment and can evaluate bladder and rectum repletion. This technique should be compared with fiducial markers. Barney, et al. [25] compared IGRT with fiducial markers and CBCT for daily localization of prostate cancer in 36 patients and found 81 of 286 treatments (28%) resulted in a > 5.0 mm difference in one or more dimensions. The most important shift disagreements (> 5 mm) were in the AP (27.6%) and the SI (27.3%) dimensions. The authors explained their results by gland deformation or motion due to bladder or rectal filling between portal and CBCT imaging, slight patient motion between image acquisitions, and the difficulty in obtaining a reproducible and accurate delineation of the prostate with CBCT due to the quality of the images and the uncertainty of delineation. However, CBCT is a good technique to verify bladder and rectum repletion. In this study, a protocol was carried out to assess bladder and rectum repletion; patients have to empty their bladder and drink two glasses of water one hour before treatment and to empty their rectum.

## Conclusions

Prostate motion is significant during a course of radiotherapy for localized prostate cancer. Using fiducial markers as surrogates of the gland can improve the accuracy of repositioning on the prostate compared with using bony anatomy and skin marks. Margins decreases should take into account prostate inter- and intrafraction motion, as well as rotations. In regards to our results, a decrease of margins from CTV to PTV is applied in our institution for patients implanted with fiducial markers from 10 mm to 6 mm.
